# An Overdue Brain Death Assessment of Traumatic Brain Injury Patients: Review of Malaysian Medical Council 2006 Documentations

**DOI:** 10.21315/mjms2023.30.2.18

**Published:** 2023-04-18

**Authors:** Zaitun Zakaria

**Affiliations:** 1 Department of Neurosciences, School of Medical Sciences, Universiti Sains Malaysia, Kelantan, Malaysia; 2 Department of Neurosciences, Hospital Universiti Sains Malaysia, Universiti Sains Malaysia, Kelantan, Malaysia; 3 Brain and Behaviour Cluster, School of Medical Sciences, Universiti Sains Malaysia, Kelantan, Malaysia

Dear Editor,

We read with great interest the article entitled ‘Prognostic factors of severe traumatic brain injury outcome in children aged 2–16 years at a major neurosurgical referral centre’ published by the *Malaysian Journal of Medical Sciences* (MJMS) in October 2009 (1). The authors evaluated the outcome of traumatic brain injury (TBI) in children. Considering that the mortality rate secondary to trauma remains more than half of childhood mortality, with road traffic accidents (RTA) as the most common mechanism of injury, brain death is an unavoidable topic to physicians. Brain death is a clinical diagnosis. It is a state when the function of the brain as a whole including the brainstem, is irrevocably lost. A person certified to be brain dead is dead (2).

In reference to this, we would like to highlight that in Malaysia, The Brain Death Committee was formed by the Ministry of Health in late 1992. The first Consensus Statement on Brain Death was published in 2003 (3), jointly by the Ministry of Health, Academy of Medicine of Malaysia and the Malaysian Society of Neurosciences. Three years later, the Malaysian Medical Council (MMC) published a guideline to complement and ensure the medical practitioners and members of the public are well informed about the brain death assessment (2). Until today, 17 years down the line, the guideline remains unchanged. The challenges towards diagnosing brain death in Malaysia was previously apprised by the National Transplant Resource Centre (NTRC) (4). Ekram et al. (4) reported that of patients suspected of brain death, less than 50% completed the brain death assessment. The main reasons (65.9%) were either ‘unable to correct parameters for brain death diagnosis’ or ‘proceeded to cardiac death before test can be done’. They also shed light on the short-sighted standardised system developed by the ministry to report brain deaths.

The recent World Brain Death Project gives an international standardisation on minimum standards in the diagnosis of brain death (5). The components of brain death determination consist of: i) establishing prerequisites, ii) clinical examinations, iii) apnoea test, and if required iv) ancillary test, and v) documentation of death. Furthermore, within the guidelines, the ambiguity that usually lurks around the physician’s mind when deciding whether the patient fulfilled brain death criteria is answered. For example, The Neurocritical Care Society and The World Brain Death Project have suggested two apnoea tests are required in paediatrics, and the apnoea testing targets are the same as in adults. In adults, repeat apnoea testing is not required if the first test confirms apnoea (5, 6).

The Consensus Statement on Brain Death stated that brain death assessment is to be carried out by two specialists, with at least 3 years of registered clinical experience, and trained in brain death assessment and diagnosing brain death. A different specialist will carry out the second assessment which should be performed at least 6 h after the first assessment. Currently, there are no dedicated training or courses produced by the ministry or academic institutions. Hence, the credibility of the specialist may be based on self-education, learning from other specialists or watching online training. A useful and informative website by the Neurocritical Care Society offers a paid online brain death determination course to clinicians (7).

Attention is given in the event an ancillary test is required. Ancillary testing in paediatrics is treated similar to adult patients (8). During the brain death assessment (5), if the clinical examination cannot be performed adequately and an ancillary test is necessary, two examinations are not required. In this case, the time of death is declared as the time that the ancillary test results are formally interpreted and documented by the attending specialist. The MMC recognised that there is a limitation to brain blood flow—based methods used for ancillary testing, and some hospitals in Malaysia may not have the required test. Apart from the previously recommended methods (Appendix IV of Brain Death 2003) (3), there are newer methods that may be used (8–10) to complement the assessment. When we look back at the above report by Ekram et al. (4), perhaps an ancillary test is a suitable option when the parameters are difficult to correct from the adverse effects of brain death to the cardiovascular and endocrine function.

Brain death assessment requires a standardised and consistent diagnosis and documentation. Moreover, with the recently published international standards in the diagnosis of brain death, and more acceptable ancillary tests, our Malaysian brain death guideline is overdue for revision.
